# Emotional Dysregulation, Hopelessness and Dysmorphophobic Concerns Among Hospitalized Patients with Autoimmune, Inflammatory, and Metabolic Skin Disorders

**DOI:** 10.3390/bs15030354

**Published:** 2025-03-13

**Authors:** Tonia Samela, Giorgia Cordella, Valeria Antinone, Maria Beatrice Pupa, Alessandra Vendoni Capitani, Dario Didona, Luciana Di Girolamo, Anna Rita Giampetruzzi, Damiano Abeni

**Affiliations:** 1 Clinical Psychology Unit, Istituto Dermopatico dell’Immacolata, IDI-IRCCS, 00167 Rome, Italy; g.cordella@idi.it (G.C.); v.antinone@idi.it (V.A.); 2 Clinical Epidemiology Unit, Istituto Dermopatico dell’Immacolata, IDI-IRCCS, 00167 Rome, Italy; beatrice.pupa@gmail.com; 3 Department of Human Neuroscience, Sapienza University of Rome, 00185 Rome, Italy; alessandra.vendonicapitani@uniroma1.it; 4 Rare Diseases Center, Istituto Dermopatico dell’Immacolata, IDI-IRCCS, 00167 Rome, Italy; d.didona@idi.it; 5 Clinical Dermatology Unit, Istituto Dermopatico dell’Immacolata, IDI-IRCCS, 00167 Rome, Italy; l.digirolamo@idi.it (L.D.G.); a.giampetruzzi@idi.it (A.R.G.); d.abeni@idi.it (D.A.)

**Keywords:** psycho-dermatology, emotional regulation, hopelessness, dysmorphophobia, skin diseases

## Abstract

Chronic disfiguring skin conditions profoundly affect patients’ quality of life (QoL) due to their physical, psychological, and emotional consequences. Although the presence of depression and anxiety symptomatology in dermatological patients is well established, the specific roles of emotional dysregulation, dysmorphophobic concerns, and hopelessness in this population require further investigation. This study aimed for the following: (1) to assess symptoms of emotional dysregulation, dysmorphophobic concerns, and hopelessness in hospitalized patients with severe dermatological diseases; (2) analyze whether emotional dysregulation mediates the relationship between dysmorphophobic concerns and hopelessness. A cross-sectional study was conducted with 120 hospitalized dermatology patients. Patients completed standardized measures, including the Emotional Dysregulation Scale (EDs), Beck Hopelessness Scale (BHS), and the Questionario sul Dismorfismo Corporeo “Body Dysmorphic Disorder Questionnaire” (QDC). Disease severity and pain perception were assessed using the Physician Global Assessment (PGA) and the Numerical Rating Scale (NRS). Significant associations were observed between emotional dysregulation, dysmorphophobic concerns, and hopelessness. Emotional dysregulation partially mediated the relationship between dysmorphophobic concerns and hopelessness (indirect effect: *b* = 0.013, CI [0.004, 0.026]). Higher dysmorphophobic concerns were associated with emotional dysregulation, which, in turn, predicted greater hopelessness. Emotional dysregulation seems to play a critical role in the relationship between dysmorphophobic concerns and hopelessness in dermatological patients.

## 1. Introduction

Chronic skin conditions are characterized by significant morbidity and, in severe cases, require hospitalization due to complications, relapses, or treatments that cannot be administered in outpatient settings. Psoriasis (PP), pemphigus vulgaris (PV), leg ulcers, and systemic sclerosis (SSc) are among the disfiguring chronic skin diseases that most profoundly impact both physical health and psychological well-being.

PP affects over 60 million adults and children worldwide and shows a bimodal onset at the age of 16–22 years and 55–60 years (Griffiths et al., 2021). The most common form of PP is plaque psoriasis, characterized by silver, sharp, scaly plaques that usually involve the extensor aspects of the knees and elbows, the lumbosacral region, and the scalp. Since PP involves visible areas, most patients show a reduction in their quality of life (QoL), and many feel a substantial, negative effect on their psychosocial well-being (Singh et al., 2017).

PV belongs to the group of bullous diseases, characterized by an intraepidermal skin detachment that leads to painful blisters and erosions on the skin and/or mucosae (Didona et al., 2022; Didona et al., 2019). The exact prevalence of PV is unknown (Eaton et al., 2010; Hübner et al., 2016). Mostly, PV initially manifests with extremely painful erosions of the oral mucosa, leading to dysphagia and a reduction in food intake, which dramatically impair the QoL of PV patients, more than other chronic skin diseases (Didona et al., 2022; Paradisi et al., 2009; Penha et al., 2015).

Leg ulcers often represent a manifestation of underlying systemic diseases, like diabetes mellitus and/or atherosclerosis (Dissemond et al., 2024). Although their pathogenesis and clinical features are extremely varied, leg ulcers are characterized by a chronic relapsing course that has a massive impact on the QoL of the patients (Oe et al., 2024). Furthermore, leg ulcers can lead to disfiguring scars and to global anatomic alterations due to the impaired performance of the lower extremities (Dissemond et al., 2024; Guarnera et al., 2007; Tabolli et al., 2007).

SSc is a rare autoimmune disease characterized by immune dysregulation, vascular damage, and fibrosis (Matucci-Cerinic et al., 2013). SSc shows a progressive course that can lead to severe organ impairment and painful necrotizing digital ulcerations (Arango et al., 2024). Furthermore, progressive sclerosis dramatically impacts the face and general appearance of patients, leading to major depressive episode/disorder and panic disorder (Romanazzo et al., 2024).

While the physical manifestation of these disfiguring conditions are well documented (Didona et al., 2023; Gisondi et al., 2017; Monami et al., 2023; Riemekasten & Müller-Ladner, 2024), their psychological and emotional consequences, particularly in hospitalized patients with severe forms of these diseases, need further examination. Existing research has already explored the prevalence of depression and anxiety symptomatology in this population (Jalenques et al., 2022; Patel et al., 2022; Pourali et al., 2021), but there is a need for further investigation into the underlying psychological mechanisms that may contribute to these conditions.

For example, SSc, skin ulcers, PP, and PV have a visible and stigmatizing nature, which can exacerbate dysmorphophobic concerns (Schut et al., 2022). Dysmorphophobic concerns are characterized by a preoccupation with perceived defects in appearance that lead to compensatory behaviors. These behaviors commonly include the following: (i) mirror checking, (ii) frequent comparisons of appearance to others, and (iii) attempts to camouflage the perceived flaws. Dysmorphophobic symptomatology is associated with significant psychological distress and heightened suicidality. For instance, a multicentric study by Schut et al. (2022) among 5487 dermatological outpatients and 2808 healthy controls from 17 European countries revealed that clinically relevant BDD symptoms were reported by 10.5% of the patients vs. 2.1% of the controls. Although body dysmorphic concerns are frequently found in dermatological settings, it often remains unrecognized in clinical routines (Stangier et al., 2003); also, without effective intervention, dysmorphophobic concerns tend to persist chronically, with minimal spontaneous remission (Phillips, 2015).

Moreover, the recent literature highlights a growing recognition of the interplay between chronic disfiguring skin diseases and mental health (Renna, 2021), suggesting that these conditions may predispose patients to emotional dysregulation, a state characterized by struggles in regulating emotional responses. Research on patients with chronic inflammatory skin diseases revealed that they are prone to develop psychopathological symptoms (Butt et al., 2022; Samela et al., 2022; Samela et al., 2024) comparable with those of patients affected by other severe chronic diseases (Sampogna et al., 2006). The emotional load experienced due to these conditions could be ascribable to symptoms of emotional dysregulation, among other factors, as observed in samples of patients with psoriasis (Almeida et al., 2021; Almeida et al., 2017; Ciuluvica et al., 2014; Ciuluvica et al., 2019). In fact, PP patients showed significant difficulties in accepting their emotional reactions, lacked effective emotion regulation strategies, and struggled with understanding and interpreting their emotional states (Fino et al., 2021; Innamorati et al., 2016). The presence of scaly, inflamed plaques and of the discomfort which comes with these symptoms has a strong impact on self-image. Significant changes in physical appearance, along with emotional dysregulation, can make patients highly vulnerable to body image disturbances (Schut et al., 2022). However, as far as we know, the presence of emotional regulation issues in patients with PV or SSc seems to be scarcely reported in the literature.

On the other hand, it is documented that patients with PV, SSc, or skin ulcers may be more vulnerable to body image disturbances due to the visible manifestations of disease (Fox et al., 2020). PV is characterized by painful blistering and visible erosions; SSc progression often manifest in characteristic scleroderma facies (Pelechas et al., 2023); skin ulcers, regardless of their etiology, are characterized by visible erosions of the skin. In fact, a healthy skin is important for mental well-being and self-esteem (Kottner et al., 2020), the skin being the largest and most visible part of our body; chronic inflammatory dermatological diseases affect integrity and appearance of the skin, increasing psychological distress, frustration, shame, and depression (Urpe et al., 2005). Emotional dysregulation can amplify psychological distress and contribute to maladaptive behaviors, complicating disease management. Moreover, emotional dysregulation prevents adaptive strategies for emotional regulation, fostering a depressive symptomatology (Boullion et al., 2021). Another factor, linked to emotional regulation deficits, which could lead to reduced motivation, poor adherence to treatment, and poor QoL problems, is hopelessness (Vatan et al., 2014). In particular, hopelessness is a cognitive experience characterized by a prolonged negative outlook on life, where people expect negative consequences and have a catastrophic perception of the future, along with the feeling that their current situation has a low probability of changing (Beck et al., 1974). The chronicity, relapsing nature, and substantial treatment burden associated with chronic skin diseases may further exacerbate hopelessness (Karakurt et al., 2018). Also, stressful life events, like living with a chronic disease, further exacerbate hopelessness by reinforcing negative attributions and impairing emotional or cognitive control, increasing vulnerability to mood disorders like depression (Parada-Fernández et al., 2021). Such concerns are often overlooked in clinical practice, despite their significant impact on patients’ overall QoL. Moreover, this study aims to contribute to a deeper understanding of the complex interplay between psychological factors in dermatological patients, ultimately informing the development of targeted interventions.

### Aims

Thus, in this study we aimed for the following: (i) to describe the presence of symptoms of hopelessness, emotional dysregulation, and dysmorphophobic concerns in hospitalized adult patients with severe dermatological conditions; (ii) to analyze whether dysmorphobic concerns and hopelessness were associated with a higher presence of emotional dysregulation, based on the observation that the highly disfiguring potential of skin conditions could lead to dysmorphobic concerns, and that the chronic nature of this condition could increase hopelessness, possibly through the emotional dysregulation accompanying those concerns. The findings will inform the development of targeted interventions to address both the physical and emotional needs of this vulnerable patient population, ultimately improving clinical outcomes and QoL.

## 2. Materials and Methods

### 2.1. Participants

This cross-sectional, observational study was approved by the Lazio Region Ethics Committee, Area 5th (Prot. n of approval 770/1). This research was conducted in compliance with the Helsinki Declaration. Data were collected from July to October 2024, at the Dermatology Department of the IDI-IRCCS, Rome, Italy, in which consecutive patients who agreed to participate in the study and signed the written informed consent, were enrolled. The inclusion criteria were as follows: (i) to be 18+ years of age; (ii) to have a diagnosis of a skin disease with a degree of severity requiring hospitalization (i.e., psoriasis; autoimmune bullous diseases; skin ulcers; systemic sclerosis) identified during the dermatological examination; and (iii) to have the ability to understand and complete the standardized tests required for the study. The exclusion criteria were as follows: (i) the presence of past or current diagnosed psychiatric disorders; (ii) the inability to complete the assessment for any reason, including denial of informed consent. Participants received no honorarium or any benefits from participating in the study. No participants withdrew from the study during the questionnaire completion process.

### 2.2. Instruments

A standardized form was completed by clinicians regarding diagnosis and disease severity through the Physician Global Assessment (PGA). Participants completed a standardized form with sociodemographic information (e.g., age; sex; weight and height in order to calculate the Body Mass Index (BMI); marital status; education level). Participants were also administered the Italian versions of the Emotional Dysregulation Scale (EDs), the Beck Hopelessness Scale (BHS), and the Questionario sul Dismorfismo Corporeo, “Body Dysmorphic Disorder Questionnaire” (QDC).

The PGA (Gottlieb et al., 2019; Pascoe et al., 2015) is a widely used 5-point scoring system used to assess disease severity in dermatology, particularly in inflammatory skin disorders; it allows comparisons between different conditions. Pain perception was assessed through the Numerical Rating Scale (NRS). The NRS is a psychometric instrument designed to assess the level of pain related to symptom severity in patients, and it is used to achieve a statistically measurable and reproducible classification of symptom severity and disease control. For this study, the scale was set on a 10-point Likert scale.

The Italian version of the EDs (Powers et al., 2015; Raimondi et al., 2022) is a 12-item self-report one-factor measure which assesses emotional dysregulation (e.g., “Emotions overwhelm me”), cognitive domains (e.g., “When I am feeling bad, I have trouble remembering anything positive, everything just seems bad”), and behavioral domains (e.g., “When my emotions are strong, I often make bad decisions”). Participants rate each item on a 7-point Likert scale, with higher scores indicating greater emotional dysregulation. EDs has also been successfully applied in various clinical populations (Christ et al., 2019; Pencea et al., 2020). The EDs’ utility within a hospital setting is enhanced by its concise item count, facilitating efficient administration and reducing respondent burden.

The Italian version of the BHS (Beck et al., 1974; Innamorati et al., 2014) is a 20-item self-report dichotomic (Yes/No) scale measuring negative attitudes about the future. In the construction of the scale, there are 11 items for which the individual has to endorse a pessimistic statement and 9 items for which the individual has to deny an optimistic statement. The items were keyed in this way in order to prevent possible response sets and careless responding. The Italian version of the questionnaire, validated for hospitalized patients, has one factor structure with one total score. Scores range between 0 and 20, with higher scores indicating greater hopelessness. Prior research has utilized it in dermatological populations, supporting its applicability (Hamidizadeh et al., 2020; Ortiz-Álvarez et al., 2023; Sayar et al., 2001). The Italian version demonstrates robust psychometric properties for medical patients (Innamorati et al., 2014).

The QDC (Cerea et al., 2017) is a 40-item, self-report, one-factor questionnaire designed to assess the full spectrum of Body Dysmorphic Disorder (BDD) phenomenology and its associated clinical features in Italian samples. Based on the extensive BDD literature, the QDC captures core BDD symptoms, such as repetitive behaviors (e.g., mirror checking, excessive grooming), mental acts (e.g., negative body comparisons), and avoidant behaviors. Additionally, it evaluates the severity of BDD symptoms using a 7-point Likert scale and explores the impact of BDD symptomatology on QoL, including the pursuit of cosmetic procedures, and suicidal ideation. A higher score is indicative of higher dysmorphophobic concerns. Unlike other self-report BDD measures, the QDC provides a comprehensive assessment of the disorder, addressing its multifaceted nature, in line with the scope of our study.

### 2.3. Statistical Analysis

All the analyses were performed with the Statistical Package for the Social Sciences (SPSS) 28.0 (IBM Corp., Armonk, New York, USA, 2021). All statistics were considered significant at *p* < 0.05. Questionnaires with missing data were excluded from the final analyses. However, concerning the completed self-reported questionnaires, when sociodemographic characteristics were missing, these data were left as missing, as stated in Table 1.

Categorical variables were described as numbers and percentages, and continuous variables were firstly classified into different levels and then described as discrete. Cronbach’s alpha was computed as a measure of reliability for self-report questionnaires. One-way ANOVA was performed to evaluate the possible presence of significant differences in the mean score of self-report questionnaires obtained by patients among the 4 different dermatologic diagnoses; Pearson’s correlation coefficient was used to assess the relationships between the variables of interest.

A simple mediation model with a single mediator (model no. 4) was tested, using the PROCESS V3.5 macro for SPSS (Hayes & Preacher, 2013). We report unstandardized coefficients (b), and their 95% bootstrap confidence intervals (95% CIs) (with 5000 bootstrap samples) (Preacher & Hayes, 2008). According to Preacher and Hayes, confidence intervals that do not include zero could provide evidence of a significant indirect effect. Even though mediation models cannot identify causal relationships between variables when data from correlational studies are used (Maxwell et al., 2011), these techniques can be effective in analyzing whether a third variable could mediate the relationship between two other variables (Salthouse, 2011).

## 3. Results

One-hundred twenty patients voluntarily participated in the study and were assessed during the period of their hospitalization between July 2024 and October 2024. The mean age of the participants was 58.6 years (SD = 15.9; range: 18–93 years). The descriptive statistics for the sociodemographic and clinical variables of the sample (N = 120) are displayed in Table 1. The median age of the participants was 60 years, with 47.9% under 60 and 52.1% over 60. Most of the sample was female (72.3%), and a small proportion of participants reported being non-smokers (22.5%) or ex-smokers (15.8%). Regarding alcohol consumption, 62.7% reported not consuming alcohol, while 9.3% reported regular consumption, and 28.0% reported occasional consumption. The average disease duration was 10.2 years (±10.4 years), and the average education level was 12.4 years (±4.2 years).

All participants received information about the purpose of the study, provided written informed consent, and completed the assessment anonymously. The Cronbach α values were 0.88, 0.93, 0.88, respectively, for the BHS, EDs, and QDC scales.

To examine the influence of sociodemographic and clinical variables on BHS, EDs, and QDC scores, analyses of variance (ANOVA) and independent sample *t*-tests were conducted. ANOVA revealed no significant differences in BHS, EDs, QDC, NRS, or PGA scores across diagnostic groups: BHS, *F*(3, 114) = 1.81, *p* = 0.149; EDs, *F*(3, 114) = 0.40, *p* = 0.757; QDC, *F*(3, 114) = 2.38, *p* = 0.073; NRS, *F*(3, 107) = 0.15, *p* = 0.929; PGA, *F*(3, 63) = 0.91, *p* = 0.442. Independent sample *t*-tests highlighted significant differences between age groups (≤60 years vs. >60 years) for BHS scores, *t*(101.84) = −2.02, *p* = 0.046, 95% CI [−3.50, −0.03], and QDC scores, *t*(101.71) = 4.07, *p* < 0.001, 95% CI [15.34, 44.55], but not for EDs scores, *t*(115) = 0.75, *p* = 0.453, 95% CI [−3.83, 8.52]. No significant differences were found between males and females for BHS scores, *t*(117) = 0.32, *p* = 0.750, 95% CI [−1.65, 2.29], EDs scores, *t*(117) = 0.61, *p* = 0.547, 95% CI [−4.72, 8.86], or QDC scores, *t*(117) = 1.96, *p* = 0.053, 95% CI [−0.21, 33.58]. Furthermore, no significant differences were observed in BHS, EDs, and QDC scores based on patient socio-demographic features (marital status: “married/cohabiting/stable relationship” vs. “single/widowed/separated”; smoking status: “smokers” vs. “non-smokers”; alcohol consumption: “consumers” vs. “non-consumers”).

Concerning Pearson’s correlation, the total EDs score was significantly and moderately associated with the total BHS score (r = 0.45, *p* < 0.001), and with the total QDC score (r = 0.30, *p* < 0.001). Moreover, total BHS and EDs scores were positively and moderately associated with NRS scores (EDs r = 0.30, *p* = 0.001; BHS = 0.38, *p* < 0.001); NRS scores were not associated with QDC scores (r= 0.17, *p* = 0.070). Correlations between variables are reported in Table 2.

**Figure 1:**
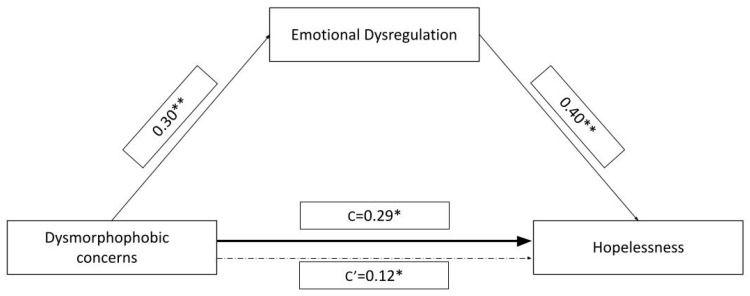
Mediation model with QDC symptomatology (dysmorphophobic concerns) as IV, BHS severity (hopelessness) as DV, and EDs (emotional dysregulation) as a mediator. Path values represent standardized regression coefficients. “c’” represents the indirect effect of QDC scores on BHS scores (confidence intervals from 5000 bootstrapping analysis are reported). “c” represents the effect of dysmorphophobic concerns on hopelessness before the inclusion of the mediating variable. * *p* < 0.05, significant effect; ** *p* < 0.001, significant effect. This figure is original, and no licenses are needed.

In the mediation model we tested, we included QDC scores as the independent variable, BHS scores as the dependent variable, and EDs scores as an independent mediator (Figure 1). The model accounted for 22.7% of the variance in BHS scores (*F*_2;117_ =17.2, *p* < 0.001). Both the direct (*c*′ = 0.019, SE = 0.009, t = 2.01, *p* = 0.046) and total effects (*c* = 0.033, SE = 0.010, *t* = 3.30, *p* = 0.001) of QDC scores on BHS scores were significant. The indirect effects of QDC scores on BHS scores via the EDs scores were also significant (*b* = 0.013, SE = 0.005, bootstrap CI [0.004, 0.026]), indicating that QDC scores influenced BHS scores indirectly via EDs scores. The analysis demonstrates that EDs scores partially mediate the relationship between QDC scores and BHS scores. Higher dysmorphophobic concerns are associated with increased emotional dysregulation symptoms, which in turn predict higher hopelessness. Although a direct relationship between QDC scores and BHS scores persists, the mediation highlights the importance of emotional dysregulation in understanding this relationship. These findings underscore the role of emotional dysregulation in shaping the relationship between dysmorphophobic concerns and hopelessness in this sample.

## 4. Discussion

This study explored the relationships between dysmorphophobic concerns, emotional dysregulation, and hopelessness in a sample of hospitalized dermatologic patients. In line with our research hypothesis, dysmorphophobic concerns were significantly and independently associated with hopelessness. The results highlight significant associations among these variables, underscoring the potential interplay between body image disturbances, emotional regulation strategies, and hopelessness in individuals who cope with severe chronic skin conditions. The existing literature has established a link between emotion regulation difficulties and several negative mental health outcomes. For example, Miranda et al. (2013) proposed a pathway in which the perceived inability to regulate emotions leads to rumination, which fuels hopelessness, ultimately increasing the risk of suicidal ideation, based on a sample of young adults. This aligns with Turton et al. (2021), who, in a systematic review, explored the complex relationship between emotion regulation and suicidal thoughts and behaviors, highlighting the roles of rumination in exacerbating hopelessness and increasing suicide risk. Their study suggested the presence of a mechanism whereby difficulties in managing emotions, particularly negative ones, can contribute to depressive symptoms and hopelessness, ultimately increasing vulnerability to suicidal behaviors. Specifically, the inability to effectively regulate negative affect may lead to a greater intensity and duration of negative emotional experiences, fueling feelings of despair and hopelessness. This cascade of negative emotional states can then increase the likelihood of suicidal thoughts and attempts. Although individuals with BDD and chronic skin conditions frequently experience overlapping psychological distress, the complex relationship between these conditions, including the potential for comorbidity, symptom overlap, and shared underlying vulnerabilities, warrants further study (Beck et al., 1974; Boullion et al., 2021; Flores-Kanter et al., 2019).

Also, based on our results, difficulties in regulating emotions may contribute to both a heightened focus on perceived flaws in appearance and a pessimistic attitude concerning the future in dermatologic patients. Specifically, individuals with heightened dysmorphophobic concerns appeared more likely to experience emotional dysregulation, which, in turn, was associated with increased hopelessness. While a direct association between dysmorphophobic concerns and hopelessness was observed, the indirect pathway acting through emotional dysregulation highlights the potential importance of emotional processes in understanding this relationship. This phenomenon seems to align with the concept of emotional reasoning. The theory of emotional reasoning describes the psychological mechanism through which human beings tend to use their emotions as significant information to express evaluations about the world rather than to refer objectively to reality (Gangemi et al., 2021). It is possible that symptoms could amplify emotions, which, in turn, could exacerbate symptoms. Thus, it is possible to assume that people with a negative self-perception due to their skin condition may develop greater hopelessness and dysmorphic concerns, creating a reinforcing cycle.

These findings partially align with those of prior research, suggesting that emotional dysregulation could be considered as a risk factor for psychiatric vulnerability (Cole et al., 2017), potentially amplifying psychological distress (Hofmann et al., 2012) and exacerbating negative thought patterns (Yalvaç & Gaynor, 2021). The model tested in this study accounted for approximately 23% of the variance (Figure 1) in hopelessness scores, indicating that while these factors are relevant, additional variables likely contribute to hopelessness in this patient population. Furthermore, the lack of significant differences in mean scores for QDC, EDs, and BHS across patient groups (i.e., psoriasis, systemic sclerosis, pemphigus vulgaris, and skin ulcers) suggests that these psychological constructs may operate similarly in these different medical conditions.

In our study, hopelessness and emotional dysregulation were also moderately associated with pain severity, as measured by the NRS, highlighting a potential link between psychological distress and the subjective experience of physical symptoms. This association is consistent with the existing literature about pain experience in patients with other chronic conditions like HIV/AIDS (Rogers et al., 2021) or chronic back pain (Overstreet & Goodin, 2018). This association, however, was not observed between dysmorphophobic symptoms and pain severity, which may indicate that these concerns are less influenced by physical sensations and more rooted in cognitive and emotional processes. It is possible to hypothesize that the lack of a significant association between dysmorphophobic symptoms and pain severity may be explained by the prevalent focus of dysmorphophobia on cognitive and emotional processes related to perceived appearance flaws, rather than direct physical sensations. In fact, individuals with dysmorphophobia tend to be intensely preoccupied with perceived defects, leading to heightened self-focus and a distorted body image.

This cognitive preoccupation may overshadow or diminish the awareness and reporting of physical pain sensations. Essentially, is possible to hypothesize that their attention is directed towards the perceived flaw, not towards other bodily sensations, including pain. Also, while our findings did not reveal a significant association between dysmorphophobic symptoms and pain severity, this does not preclude the possibility of a more complex relationship between them. In fact, it is also possible that the specific types of pain experienced, the location of pain, or the coping strategies employed by individuals with dysmorphophobic concerns could influence this association. Future research employing more detailed measures of pain and exploring potential moderating factors, such as specific cognitive and emotional regulation strategies, would be valuable in further elucidating this relationship. From a clinical perspective, our study underscores the potential utility of addressing emotional dysregulation in order to mitigate hopelessness, particularly in patients with significant body image concerns. The use of maladaptive defense mechanisms, common in psychosomatic presentations, hinders emotional awareness and the ability to manage emotional intensity (Settineri et al., 2019). Considering the significant correlations between emotional dysregulation and dysmorphophobic concerns, as well as hopelessness, it is plausible that these maladaptive mechanisms play a crucial role in the cognitive and emotional processes associated with these conditions. For example, the use of denial or avoidance might prevent individuals from acknowledging and processing their emotions, leading to heightened distress and a distorted perception of their body image.

### 4.1. Clinical and Research Implications

Interventions that target emotion regulation may help patients in managing emotional reactivity and reduce psychological distress (Saccaro et al., 2024).

Specifically, cognitive reappraisal, such as identifying and challenging maladaptive thought patterns related to skin condition or body image (Mulgrew & Boyer, 2024), could be implemented. This approach can help patients reframe negative thoughts and reduce emotional reactivity (Orvell et al., 2021) to perceived flaws or skin symptoms.

Furthermore, mindfulness-based interventions, which emphasize present moment awareness and non-judgmental acceptance of emotions, may be particularly helpful. Mindfulness practices, such as distress tolerance training and/or self-compassion strategies, can help patients in observing their negative thoughts and emotional responses without automatically reacting to them, fostering a greater sense of emotional control (Clarke et al., 2022; Hoge et al., 2021). These skills can provide patients with concrete strategies for coping with difficult emotions in the moment, reducing reliance on potentially maladaptive coping mechanisms, and could help people adjust to chronic skin conditions (Clarke et al., 2022). By equipping patients with these tools, they can be empowered to take control of their emotional experience, leading to a reduction in psychological distress and improved QoL. While our findings are based on a hospitalized sample with a long disease duration, it is plausible that similar relationships between emotional dysregulation, hopelessness, and dysmorphophobic concerns may exist in outpatient settings. This raises the potential for extending the use of these measures for early screening and preventative interventions in individuals with dermatological conditions who may not require hospitalization. In fact, early identification of emotional dysregulation and body image concerns could facilitate prompt intervention, potentially mitigating the development of hopelessness and improving overall psychological well-being. However, further research is needed to specifically investigate the applicability and effectiveness of these measures in non-hospitalized populations and to explore the feasibility of implementing screening and preventative interventions within diverse clinical contexts.

### 4.2. Limitations

These results remain preliminary and require further investigation. One limitation is the cross-sectional design of this study, which precludes conclusions about causality; longitudinal studies are needed to confirm the directionality of the relationships between the variables of interest. The second limitation is the study’s focus on older patients with longer disease durations; this may limit generalizability to younger patients or those with less severe conditions. This is supported by findings of increased hopelessness in older participants and higher dysmorphophobic concerns in younger ones, highlighting the potential impact of age on psychological outcomes.

Additionally, unmeasured variables, such as perceived stigma or social support, may play a role in these relationships and warrant further exploration. Future research should aim to replicate these findings in larger and more diverse samples, examining the interplay of these factors over time.

In summary, while cautious interpretation is necessary given the study’s limitations, these findings suggest avenues for further research on and clinical intervention for the role that emotional dysregulation may play in the relationship between dysmorphophobic concerns and hopelessness in hospitalized dermatologic patients.

## 5. Conclusions

In conclusion, this study provides preliminary evidence about the interplay between dysmorphophobic concerns, emotional dysregulation, and hopelessness in hospitalized dermatologic patients. Our findings suggest a significant and independent association between dysmorphophobic concerns and hopelessness, mediated by difficulties in emotional regulation. This could suggest that difficulties in regulating emotions not only exacerbate dysmorphophobic symptoms but also contribute to a pessimistic outlook, potentially through mechanisms such as emotional reasoning and the use of maladaptive defense mechanisms. The observed associations between emotional dysregulation and both dysmorphophobic concerns and hopelessness underscore the clinical significance of addressing emotional processes in this patient population. Notably, the moderate associations between hopelessness, emotional dysregulation, and pain severity, coupled with the absence of a direct link between dysmorphophobic concerns and pain, highlight the predominantly cognitive and emotional nature of dysmorphophobia. Thus, it is possible to hypothesize that interventions targeting cognitive reappraisal, mindfulness, and distress tolerance skills may be beneficial in mitigating psychological distress and improving QoL in these patients. While our model accounted for a significant portion of the variance in hopelessness, the remaining variance suggests the involvement of other potentially influential factors. Future research should explore these additional variables, as well as examine the potential moderating effects of specific pain types, locations, and coping strategies. Finally, this study contributes to a growing body of the literature highlighting the importance of integrated psychological interventions in the management of dermatologic conditions, particularly those characterized by body image concerns and emotional dysregulation.

## Figures and Tables

**Table 1:** Descriptive statistics for sociodemographic and clinical variables in the sample.

	Frequency	%
Overall	120 *	100.0
Age (median)		
<60	56	47.9
60+	61	52.1
Sex		
Female	86	72.3
Male	33	27.7
Smoke		
Yes	74	61.7
No	27	22.5
Ex-smokers	19	15.8
Alcohol		
No	74	62.7
Yes	27	9.3
Occasionally	19	28.0
Dermatological diagnosis		
Psoriasis	30	25.0
Pemphigus vulgaris	34	28.3
Skin ulcers	11	9.2
Systemic sclerosis	45	37.5
	mean	SD
Disease duration (years)	10.2	(±10.4)
Education levels (years)	12.4	(±4.2)
BHS	5.6	(±4.8)
EDs	33.1	(±16.7)
QDC	83.4	(±42.1)
NRS for pain	5.2	(±2.7)
PGA	2.9	(±0.8)

* Total may vary due to missing data; SD = standard deviation; BHS = Beck Hopelessness Scale; EDs = Emotional Dysregulation Scale; QDC = Questionario sul Dismorfismo Corporeo; NRS = Numerical Rating Scale; PGA = Physician Global Assessment.

**Table 2:** Pearson correlations between measures of emotional dysregulation, dysmorphophobic concerns, hopelessness, and sociodemographic features of the sample (N = 120).

	BHS Score	QDC Score	EDS Score	Sex	Age	Disease Duration	NRS	PGA	Marital Status	Education (Years)	BMI
BHS score	--										
QDC score	0.29 **	--									
EDS score	0.45 **	0.30 **	--								
Sex	−0.03	−0.18	−0.06	--							
Age	0.23 *	−0.37 **	−0.02	0.13	--						
Disease duration	0.24 *	0.09	0.06	−0.12	−0.11	--					
NRS for pain	0.38 **	0.17	0.30 **	−0.13	0.26 **	0.10	--				
PGA	0.19	0.14	0.04	0.09	0.26 *	0.04	0.56 **	--			
Marital status	0.03	−0.14	0.03	−0.08	0.42 **	0.01	0.11	0.01	--		
Education (years)	−0.22 *	−0.05	−0.12	0.11	−0.27 **	0.02	−0.37 **	−0.12	−0.18 *	--	
BMI	0.03	0.12	0.11	0.20 *	−0.05	−0.02	0.03	−0.11	−0.05	−0.04	--

* The correlation is significant at the 0.05 level; ** The correlation is significant at the 0.01 level. BHS = Beck Hopelessness Scale; EDs = Emotional Dysregulation Scale; QDC = Questionario sul Dismorfismo Corporeo; NRS = Numerical Rating Scale; PGA = Physician Global Assessment; BMI = Body Mass Index.

## Data Availability

The data that support the findings of this study are available from the corresponding author, TS, upon reasonable request.
